# Transcriptome analysis of liver and ileum reveals potential regulation of long non-coding RNA in pigs with divergent feed efficiency

**DOI:** 10.5713/ab.24.0434

**Published:** 2024-10-25

**Authors:** Zhenjian Zhao, Shujie Wang, Kai Wang, Xiang Ji, Dong Chen, Qi Shen, Yang Yu, Shendi Cui, Junge Wang, Ziyang Chen, Guoqing Tang

**Affiliations:** 1 Key Laboratory of Livestock and Poultry Multi-omics, Ministry of Agriculture and Rural Affairs, College of Animal Science and Technology, Sichuan Agricultural University, Chengdu 611130, China; 2 Farm Animal Genetic Resources Exploration and Innovation Key Laboratory of Sichuan Province, Sichuan Agricultural University, Chengdu 611130, China

**Keywords:** Competing Endogenous RNA (ceRNA) Network, Feed Efficiency, Long Non-Coding RNA (lncRNAs), Pig, Transcriptome Analysis

## Abstract

**Objective:**

Long non-coding RNA (LncRNA) plays a significant role in regulating feed efficiency. This study aims to explore the key lncRNAs, associated genes, and pathways in pigs with extreme feed efficiencies.

**Methods:**

We screened pigs with extremely high and low residual feed intake through a 12-week animal growth trial and then conducted transcriptome analysis on their liver and ileum tissues. We analyzed the differential expressed lncRNAs, microRNAs (miRNAs), and messenger RNAs through target gene prediction and functional analysis. And we identified key lncRNAs and their potential regulatory genes associated with feed efficiency through the construction of competitive endogenous RNA network.

**Results:**

Differentially expressed lncRNAs were pinpointed in the liver, revealing 23 crucial target genes primarily associated with guanosine triphosphate metabolism and glycolipid biosynthesis. In the ileum, a screening identified 92 pivotal target genes, mainly linked to lipid and small molecule metabolism. Moreover, LOC106504303 and LOC102160805 emerged as potentially significant lncRNAs respectively, playing roles in mitochondrial oxidative phosphorylation in the liver, and lipid and cholesterol metabolism in the ileum.

**Conclusion:**

The lncRNAs regulate energy metabolism and biosynthesis in the liver, and the digestive absorption capacity in the small intestine, affecting the feed efficiency of pigs.

## INTRODUCTION

Feed efficiency is a crucial economic trait that plays a direct role in the profitability of pig farming operations. Enhancing feed efficiency not only leads to cost reduction and increased profits but also contributes to mitigating environmental pollution [1]. Feed efficiency can be evaluated through indicators such as feed conversion ratio (FCR) or residual feed intake (RFI). RFI quantifies the discrepancy between actual and predicted feed intake [2]. A higher actual feed intake compared to the predicted intake suggests lower digestive and absorptive capacities, resulting in elevated RFI levels. RFI serves as a comprehensive metric that encompasses facets of pig physiology, including digestion, absorption, metabolism, and immunity, underpinned by intricate gene expression and regulatory networks necessitating further investigation. Notably, recent omics studies in pigs have identified key pathways influencing feed efficiency, such as carbohydrate and lipid metabolism [3–5], energy metabolism [6–8], and immune response [9–11].

Non-coding RNA (ncRNA) refers to transcribed RNA molecules that do not encode proteins but play diverse regulatory roles in various biological processes, such as the modulation of DNA epigenetic modifications, transcription, and post-transcriptional gene expression [12]. MicroRNAs (miRNAs), which exert gene expression control by binding to target messenger RNA (mRNA), leading to the degradation or inhibition of the targeted transcript, are present in all tissues and most cell types and are involved in crucial biological processes, including livestock feed efficiency [13]. Mitochondria and energy metabolism are closely linked to pig feed efficiency and represent key pathways regulated by miRNAs in porcine skeletal muscle. Studies have associated miR-338 [14], miR-335 [15], and miR-144 [16] with skeletal muscle mitochondria and energy metabolism [17]. In the liver of pigs, several miRNAs have been implicated in the metabolism of glucose, lipids, and proteins. For instance, miR-34a, miR-326, miR-1, and miR-185 have been recognized for their involvement in glucose and lipid metabolism [18–20].

Long non-coding RNA (lncRNA), typically exceeding 200 bases in length, can modulate gene expression through various mechanisms, including DNA methylation, histone modification, alteration of promoter activities through nucleosome repositioning, and epigenetic silencing and repression [13]. LncRNAs can also function as precursors for miRNA and act as “sponges,” binding to miRNAs and hindering their inhibitory effects on mRNA expression. Importantly, lncRNAs play a significant role in regulating feed efficiency and related traits. Hou et al [21] performed transcriptome sequencing of the skeletal muscle of high and low RFI Duroc pigs and identified 43 differentially expressed lncRNAs (DELs). Functional prediction analysis of these lncRNAs revealed their potential roles in regulating lipid metabolism, cell proliferation, and cell adhesion processes that could impact feed efficiency. Nine candidate lncRNAs were pinpointed as potential biomarkers for RFI. In a study on sheep RFI, Zhang et al [22] identified 10 key lncRNAs. Among them, LNC_000890 was suggested to regulate liver tissue metabolic efficiency and exhibited a significant association with feed efficiency by co-expressing with the ADRA2A gene. In cattle, 126 lncRNAs have been linked to feed efficiency, with 71 of them identified as pivotal lncRNAs capable of regulating the expression of mRNAs associated with feed efficiency [23].

While numerous ncRNAs have been linked to feed efficiency in pigs, existing studies have primarily delved into the roles of specific tissues and a limited array of ncRNAs, leaving a gap in comprehensive research targeting multiple tissues. Moreover, there’s a scarcity of research investigating the involvement of lncRNAs in feed efficiency across diverse tissues. Hence, our study aims to bridge this gap by conducting a joint analysis of lncRNAs affecting feed efficiency in both the liver and small intestine of pigs. Through transcriptome sequencing, we examined the differential expression of lncRNAs and miRNAs in pigs exhibiting extremely high and low feed efficiency. Subsequent target gene prediction and functional enrichment analysis helped identify relevant lncRNAs implicated in regulating feed efficiency. Additionally, by integrating mRNA and miRNA expression data, we analyzed transcriptome regulatory networks to gain deeper insights into the regulatory roles of lncRNAs in pig feed efficiency.

## MATERIALS AND METHODS

### Animals

This study utilized 209 Duroc sows obtained from a pig farm in Sichuan, China. The sows were randomly assigned to 20 pens, each housing 10 to 12 pigs and equipped with Feed Intake Recording Equipment (Osborne, Osborne Industries Inc., Osborne, KS, USA). Electronic ear tags were provided for individual identification at the feeding station, where data on tags, pre- and post-feeding weights, feed intake, and feeding duration were automatically recorded. The test pigs started at an average age of 93 days and 33.6 kg, reaching an average age of 177 days and 111 kg after a 12-week observation period. The back fat thickness (BFT) between the sixth and seventh ribs was measured using B-mode ultrasound (MyLabX7, ESAOTE, Genova, Italy) at the end of the study. The pigs were fed a commercial corn-soybean diet tailored to their body weight, following the Chinese standard GB/T 5915-2020, and were not administered any antibiotics or pharmaceuticals. Environmental conditions were meticulously controlled for comfort, with free access to clean water. Regular health assessments by veterinarians were conducted throughout the research period. Weekly calibration of the weight and feed scales on the Osborne feeding station ensured data precision.

All experimental procedures were performed by the Institutional Review Board (IRB14044) and the Institutional Animal Care and Use Committee of the Sichuan Agricultural University under permit number DKY-B20140302.

### Residual feed intake calculation and sample collection

Firstly, we conducted quality control on the recorded feeding data for each pig, ensuring accuracy within specified ranges: daily feed intake ranging from 0.5 kg to 4.5 kg, daily feeding frequency of 2 to 20 times, and daily feeding duration between 5 minutes and 2 hours. Subsequently, we calculated the initial body weight (W1), final body weight (W2), average daily feed intake (ADFI), and average daily gain (ADG) for each pig. The RFI value was then estimated using linear regression of ADFI on average metabolic body weight (AMW), ADG from 30 to 115 kg, and back fat [24]. The formula is:


RFI (g)=ADFI (g)-0.9916ADG-22.7719BFT-78.4455AMW

AMW was calculated as (*W*2^1.6^−*W*1^1.6^)/[1.6×(*W*2−*W*1)] [25].

Based on the RFI values, four pigs with extremely high RFI and four with extremely low RFI were grouped as HR and LR respectively. All eight selected pigs from the HR and LR groups were slaughtered following the Live Pig Slaughter Guidelines (GB/T 17236-2019). Prior to slaughter, pigs were starved overnight but provided unlimited water. Carbon dioxide stunning was administered before slaughter. The ileal mucosa and liver were aseptically separated and immediately transferred into sterile 15 mL cryovials, then frozen in liquid nitrogen for storage.

### RNA sequencing and identification of lncRNAs and miRNAs

RNA was extracted according to the TRIzol reagent extraction instructions. The quality of total RNA was detected by RNA-specific agarose electrophoresis and Agilent 2100. Qualified RNA was sequenced by strand-specific library construction in BGI Company, and the sequencing platform was BGISEQ-500. Small RNA and lncRNA sequencing were performed using the Illumina HiSeq 4000 platform (Illumina, San Diego, CA, USA).

FastQC was used for quality control of raw data and filtered using SOAPnuke. Clean reads were then mapped to the reference genome Sscrofa 11.1 using Hisat2. The raw reads from the lncRNA library were processed using a Perl script to remove reads containing more than 10% unknown nucleotides and low-quality reads. Then, Bowtie2 was used to map these clean data to the reference genome of pig (Sus scrofa 11.1) for lncRNA identification. The raw reads from the small RNA library were initially processed using custom Perl and Python scripts to remove redundant regions. All the unique data were aligned against the small RNAs in the Rfam (11.0) and GeneBank databases (Release 209.0) to identify and remove tRNA, rRNA, snoRNA, snRNA, and scRNA. Then, the remaining clean tags were aligned against the reference genome (Sus scrofa 11.1), and miRBase database (Release 21) was used to identify known miRNAs. Unmapped reads were aligned against other species. The identification of novel miRNAs was based on the genomic location and hairpin structure of miRNAs, using Mireap software (v0.2).

### Identification of differentially expressed lncRNAs and miRNAs

Expression values for lncRNAs, mRNAs, and miRNAs were normalized by reads per million in edgeR. Differential expression analysis was performed by DEGseq2 to identify differentially expressed RNAs. Differentially expressed mRNA (DEMs), DELs and differentially expresse miRNAs (DEMis) were defined as |log2FC| >1 and FDR<0.05.

### Functional analysis of target genes

LncRNAs play a crucial role in transcriptional regulation by both cis-regulating neighboring target genes and trans-regulating distal target genes. To identify cis-regulation, we searched for protein-coding mRNAs located within 100k upstream and downstream of each lncRNA. To identify trans-regulation, we employed RNAplex to screen for trans-acting target genes. RNAplex predicts RNA-RNA interactions using specific parameters, including a maximum seed length of 10 nucleotides and a minimum energy threshold of −30 kcaL/moL. To validate the predicted interactions, we conducted a co-expression analysis, assessing the relationship between mRNA and lncRNA expression levels using the Pearson correlation coefficient (PCC). Target genes were selected based on their expression levels and those with an absolute PCC value greater than 0.9 were chosen for further analysis.

Considering that DEMs may be directly or indirectly regulated by lncRNAs, we searched for overlapping genes between DEMs and the target genes of DELs. To explore the functionality of DELs, GO (gene ontology) and KEGG (Kyoto encyclopedia of genes and genomes) pathway analyses were performed on the overlapping genes. GO terms and KEGG pathways with a p-value less than 0.05 were considered significantly enriched.

### Construction of ceRNA interaction network

To explore the “sponge” function of lncRNAs, we constructed lncRNA-miRNA-mRNA interaction network. Firstly, we used TargetScan (v7.0), miRanda (v3.3), and MIREAP (v0.2) to predict the target genes of DELs and DEMs. Secondly, we calculated the PCC between DEMs and their target genes. Relationship pairs (miRNA-lncRNA and miRNA-mRNA) with a PCC less than −0.6 were selected as candidate pairs. Thirdly, we calculated the expression correlation between DELs and DEMs using PCC and selected relationship pairs with a PCC greater than 0.9 as candidate competitive endogenous RNA (ceRNA [lncRNA-mRNA]) pairs. Finally, GO and KEGG enrichment analyses were performed on the genes involved in the ceRNA network, and the ceRNA network was visualized using the Cytoscape software (http://www.cytoscape.org/). GO terms and KEGG pathways with a p-value less than 0.05 were considered significantly enriched.

## RESULTS

### Animal performance and feed efficiency

The 209 Duroc sows grew from an average weight of 30 kg to 115 kg on a commercial pig feed. ADFI and BFT were measured and the ADG, FCR and RFI were calculated. From this data, two sub-groups were selected with low or high RFI. The group with low RFI (−0.270±0.020) exhibited lower FCR (1.971±0.104) and ADFI (1.863±0.125) compared to the high RFI group (Table 1). However, there was no significant difference in ADG between the two groups. Interestingly, pigs with low RFI also had thinner BFT. This could suggest that pigs with low RFI more efficiently utilize the ingested energy and do not store it as subcutaneous fat in the back.

### LncRNA and miRNA sequencing data

Since ncRNAs play essential functions in post-transcriptional regulation, we selected 8 pigs with extremely high (HR, n = 4) and low (LR, n = 4) RFI and performed lncRNA and miRNA sequencing on the liver and ileum to screen important lncRNA and miRNA. For lncRNA sequencing, an average of 85,919,882 raw reads were generated per sample. After filtering and alignment, a total of 11,687 lncRNAs were identified. For small RNA sequencing, an average of 14,367,703 raw reads were generated per sample. In the end, we detected a total of 577 miRNAs, including 361 known miRNAs and 216 novel miRNAs.

### Differentially expression of lncRNAs and miRNAs

**Figure 1:**
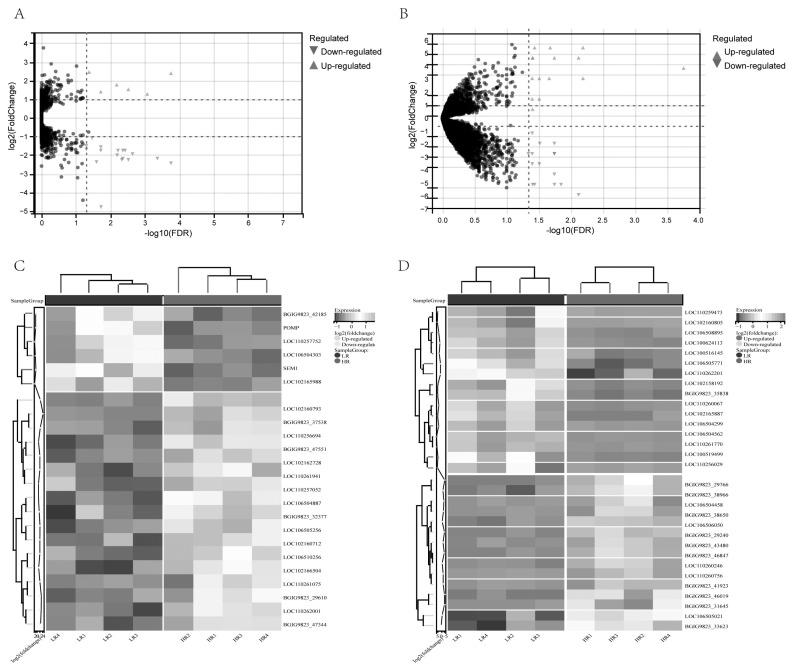
Significant differentially expressed lncRNAs between LR and HR groups. (A) Volcano plot of differentially expressed lncRNAs in the liver tissue. (B) Volcano plot of differentially expressed lncRNAs in the ileum tissue. (C) Heatmap of differentially expressed lncRNAs in the liver tissue under clustering conditions in the liver tissue. (D) Heatmap of differentially expressed lncRNAs in the ileum tissue under clustering conditions. lncRNA, long non-coding RNA; LR, low residual feed intake; HR, high residual feed intake.

The liver and ileum are important digestive organs that directly relate to pig feed utilization efficiency. Thus, we analyzed the differential expression of lncRNAs and miRNAs between the HR and LR pig groups. In liver tissue, we identified 23 significantly DELs, with 6 up-regulated and 17 down-regulated in the LR group compared to the HR group (Figure 1A). Similarly, in ileum tissue, we found 31 significant DELs, with 16 up-regulated and 15 down-regulated in the LR group compared to the HR group (Figure 1B). Clustering heatmaps (Figures 1C, 1D) displayed clear separation between HR and LR groups, affirming the reliability of our differential expression results.

We also examined theDEMis in both tissues between the LR and HR groups. Our analysis identified 361 known porcine miRNAs, with 341 in liver tissue and 356 in ileal mucosa. In the liver, we identified 23 significantly different miRNAs, with 9 up-regulated and 14 down-regulated. In the ileal mucosa, we found 72 significantly different miRNAs, with 30 up-regulated and 42 down-regulated.

### Prediction and functional analysis of lncRNA target genes in liver tissues

In the liver tissue of the LR group, we identified significantly upregulated and downregulated DELs. Notably, BGIG9823_ 28378 and BGIG9823_37538 exhibited the most significant upregulation and downregulation, respectively, in the LR group (Table 2). To elucidate the functions of lncRNAs further, we predicted the cis-regulated and trans-regulated target genes of these lncRNAs. Combining these two gene sets and eliminating duplicates, we identified 253 potential target genes regulated in cis and 527 potential target genes regulated in trans. We refined the candidate target genes of DELs by identifying overlapping regions with DEMs, resulting in the identification of 23 overlapping genes.

**Figure 2:**
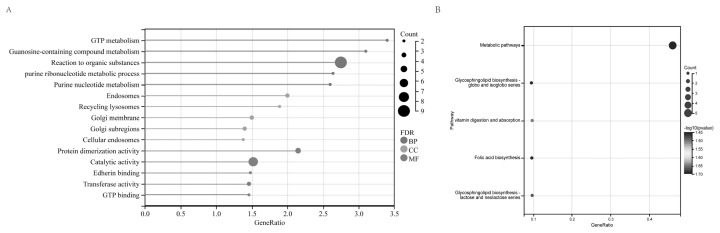
GO and KEGG enrichment results of predicted target genes of DELs in liver tissue. (A) Gene ontology analysis on the predicted target genes of DELs. (B) KEGG pathway enrichment analysis on the target genes of DELs. GO, gene ontology; KEGG, Kyoto encyclopedia of genes and genomes; DELs, differentially expressed long non-coding RNAs.

GO enrichment analysis classified the functions of the overlapping genes, revealing 100 significantly enriched GO terms (p<0.05, count>2). These enriched terms indicate that the biological processes (BP) primarily involve metabolic processes of guanosine triphosphate (GTP) and purine nucleoside-containing compounds, as well as responses to organic substances. They also encompass metabolic processes of purine ribonucleosides and purine ribonucleotides. In terms of molecular function (MF), significant enrichments suggest involvement in protein dimerization activity, catalytic activity, calcium ion binding, transferase activity, and GTP binding. Regarding cellular components (CC), significant enrichments indicate the presence of these processes in the endosome, recycling endosome, Golgi membrane, Golgi apparatus lumen, and early endosome (Figure 2A). KEGG pathway enrichment analysis revealed several pathways, including metabolic pathways, glycosphingolipid biosynthesis of the globo and isoglobo series, vitamin digestion and absorption, folate biosynthesis, as well as glycosphingolipid biosynthesis of the lacto and neolacto series (Figure 2B).

### Prediction and functional analysis of lncRNA target genes in ileum tissues

In the ileum of the LR group, LOC106504562 exhibited the most significant upregulation, while BGIG9823_46019 showed the most significant downregulation among the DELs (Table 3). To further elucidate the functions of these lncRNAs, we predicted their cis-regulated and trans-regulated target genes. Moreover, we identified 92 overlapping genes between the target genes of DELs and the DEMs.

**Figure 3:**
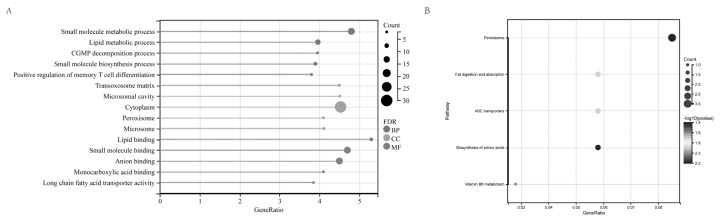
GO and KEGG enrichment results of predicted target genes of DELs in ileum tissue. (A) Gene ontology analysis on the predicted target genes of DELs. (B) KEGG pathway enrichment analysis on the target genes of DELs. GO, gene ontology; KEGG, Kyoto encyclopedia of genes and genomes; DELs, differentially expressed long non-coding RNAs.

GO enrichment analysis revealed a total of 333 significantly enriched GO terms (p<0.05, count>2). The top 5 significantly enriched terms in BP include small molecule metabolic process, lipid metabolic process, cyclic guanosine monophosphate catabolic process, small molecule biosynthetic process, and positive regulation of memory T cell differentiation. In terms of MF, the enriched terms are associated with lipid binding, small molecule binding, anion binding, monocarboxylic acid binding, and long-chain fatty acid transporter activity. Regarding CC, the enriched terms encompass the peroxisomal matrix, microbody lumen, cytosol, peroxisome, and microbody (Figure 3A). KEGG pathway analysis revealed significant enrichment in six biological pathways, including peroxisome, fat digestion and absorption, adenosine triphosphate (ATP)-binding cassette transporters, vitamin B6 metabolism, amino acid biosynthesis, and PPAR signaling pathway (Figure 3B).

### Construction of competitive endogenous RNA network

The ceRNA network plays a crucial role in the functionality of lncRNAs. We integrated transcriptome sequencing data and initially predicted miRNA-mRNA and miRNA-lncRNA target relationships using TargetScan, miRanda, and RNAhybrid, retaining only relationships present in both software. Relative expression levels from sequencing revealed a contrasting pattern between lncRNA and mRNA compared to miRNA expression, with R<−0.85 and p<0.05. Subsequently, we calculated the mRNA-lncRNA co-expression relationship and visualized the network using Cytoscape (3.9.1).

**Figure 4:**
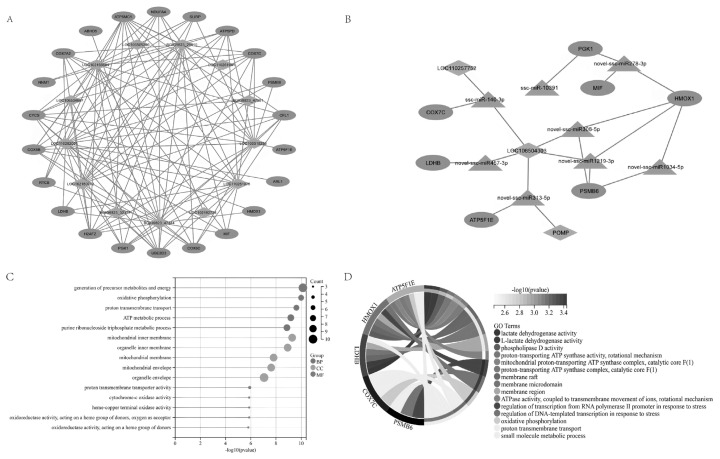
Network of lncRNAs, mRNAs and miRNAs in liver tissue. (A) Co-expression network of lncRNA and mRNA. LncRNA in green and mRNA in red. (B) Network diagram of LncRNA–miRNA–mRNA network. miRNA in blue, lncRNA in green, and mRNA in red. (C) Gene ontology analysis of genes in the interactome network. (D) Gene ontology analysis of genes in the regulatory network involving LOC106504303. lncRNAs, long non-coding RNAs; mRNA, messenger RNA; miRNAs, microRNAs.

In liver tissue of the LR group, we identified potential interactions among 22 upregulated mRNAs and 13 downregulated lncRNAs (Figure 4A). Seven lncRNAs (BGIG9823_29610, LOC106510256, LOC110261075, BGIG9823_47344, LOC102160712, LOC110262001, and LOC102166504) were found to potentially regulate the expression of multiple upregulated genes associated with mitochondria and energy metabolism (Figure 4C). Notably, LOC106504303, a core long lncRNA, was identified to regulate the expression of 5 genes through interaction with 5 miRNAs, particularly genes related to mitochondrial oxidative phosphorylation and various ATP-related enzyme activities (Figures 4B, 4D).

**Figure 5:**
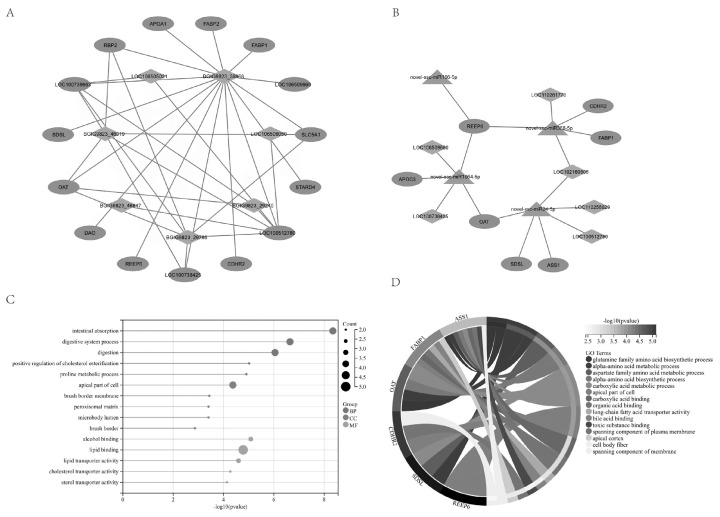
Network of lncRNAs, mRNAs and miRNAs in ileum tissue. (A) Co-expression network of lncRNA and mRNA. LncRNA in green and mRNA in red. (B) Network diagram of LncRNA–miRNA–mRNA network. miRNA in blue, lncRNA in green, and mRNA in red. (C) Gene ontology analysis of genes in the interactome network. (D) Gene ontology analysis of genes in the regulatory network involving LOC102160805. lncRNAs, long non-coding RNAs; mRNA, messenger RNA; miRNAs, microRNAs.

Similarly, in ileum tissue of the LR group, we identified 15 upregulated mRNAs and 7 downregulated lncRNAs (Figure 5A), and a triple regulatory network involving 7 upregulated mRNAs, 6 upregulated lncRNAs, and 4 downregulated miRNAs (Figure 5B). The lncRNA BGIG9823_38966 was found to regulate the expression of all 15 upregulated genes mainly associated with lipid and cholesterol metabolism (Figure 5C). Additionally, LOC102160805, highly upregulated in the LR group, was found to regulate the expression of 6 genes through interaction with 2 miRNAs, particularly genes associated with small molecule metabolism and amino acid metabolism (Figure 5D).

## DISCUSSION

Feed efficiency is a vital economic trait across livestock species such as pigs, cattle, sheep, and poultry. Feed conversion ratio (FCR) and RFI are commonly used as selection goals in animal breeding programs, making the accurate estimation of RFI crucial. RFI is calculated separately for each breed. For each animal, RFI refers to the deviation between the actual feed intake and the ADFI predicted based on maintenance and production requirements. The predicted ADFI is estimated through multiple phenotypic linear regression, which considers ADG for growth, Body Fat Thickness for the composition of weight gain, and Average Maintenance Weight for maintenance requirements. The regression models used in different studies vary slightly. For example, Gilbert et al [24] included lean meat content (LMC) in their RFI2 model, while Saintilan et al [26] incorporated both LMC and dressing percentage (DP) in their study to calculate RFI for four pig breeds. Additionally, they argue that models combining LMC and DP are more accurate. Differences in RFI estimates from various models may also lead to variations in heritability estimates, although most reported heritability values for RFI are relatively low [27–29]. In our study, we used the RFI1 model from Gilbert et al [24] to select extreme phenotypic individuals for analyzing the regulatory mechanisms and key pathways of ncRNAs on extreme RFI phenotypes. The key ncRNAs and genes we identified can serve as molecular markers for genomic breeding. Genomic selection is a reliable method for RFI selection, and these studies contribute to improving the accuracy of genomic selection.

Metabolically active tissues like the liver, muscle, adipose tissue, and small intestine have been particularly scrutinized. Liver is the central organ of whole-body metabolism [30], which directly affects the efficiency of energy conversion into tissues such as muscle and fat, thereby influencing feed efficiency. Researchers have extensively investigated the pivotal role of the liver in feed utilization and its influence on feed efficiency in pigs. These investigations encompass various aspects, including the metabolic functions of the liver, regulation of gene expression, fat metabolism, and cholesterol synthesis. Pigs with diverse phenotypes regarding feed efficiency display notable distinctions in blood parameters and levels of liver gene expression. Genes exhibiting differential expression in pigs with high feed efficiency are significantly linked to carbohydrate, lipid, and protein metabolism, indicative of heightened absorption and transportation capabilities [4,5]. Oxidative stress, inflammation, and immune responses can disrupt normal liver function and affect metabolic status, thereby impacting feed efficiency. These factors can impede food digestion, absorption, and metabolism through diverse pathways. Oxidative stress and inflammation may compromise intestinal barrier function, resulting in nutrient loss and intestinal damage, consequently diminishing feed efficiency. Additionally, immune responses consume energy and nutrients, influencing the body’s metabolic state and, consequently, feed efficiency. Thus, safeguarding liver health and mitigating oxidative stress, inflammation, and immune responses are imperative for enhancing feed efficiency [31,32].

In recent years, mounting evidence has linked lncRNA to metabolic, immune, and other traits in domesticated animals, potentially impacting feed efficiency [33]. Zhang et al [22] identified DELs and their potential target genes in the liver of sheep exhibiting extremely high feed efficiency. These genes were primarily enriched in energy metabolism pathways, suggesting their potential role in regulating hepatic metabolic efficiency. Similarly, in pigs with extreme feed efficiency, we identified a substantial number of lncRNAs. Among them, 133 lncRNAs were up-regulated and 68 were down-regulated in the low RFI group. Through differential gene expression analysis, we pinpointed 23 potential target genes mainly involved in GTP metabolism and binding, nucleotide metabolism, catalytic enzyme activity, and transferase activity.

To further explore the functional pathways and associated networks of lncRNA, we integrated the analysis of differentially expressed genes in the liver and constructed a lncRNA-mRNA interaction network. Within this network, we identified 22 up-regulated genes and 13 down-regulated lncRNAs. Further analysis indicated that these genes likely enhance mitochondrial energy metabolism, including ATP synthesis, proton transmembrane transport, and oxidative phosphorylation. For instance, Cytochrome c oxidase subunit 7C (Cox7c) is a component of respiratory chain complex IV [34], while ATP5F1E encodes a subunit of ATP synthase, crucial for cellular energy production [35]. Lactate dehydrogenase B participates in lactate and pyruvate interconversion for energy generation [36], and PSMB6 is involved in protein degradation and maintaining cellular protein balance [37]. The upregulation of these genes in the LR group suggests heightened feed efficiency and liver energy metabolism demand. Although the mechanisms of lncRNAs remain unclear, their upregulation in the liver indicates that their primary influence on feed efficiency likely occurs via energy metabolism pathways.

The digestive and absorptive capacity of the small intestine is closely related to feed efficiency traits. The structure of the pig’s small intestine, including microvilli, villous crypts, and intestinal mucosa, significantly affects nutrient absorption [38,39]. Previous research indicates that tight junctions and adherens junctions, components of the apical junctional complex, regulate the paracellular permeability of epithelial cells [40]. Tight junctions act as selective barriers, controlling mucosal permeability and regulating the entry of small molecules and ions into the body [38]. To understand how lncRNAs impact feed efficiency in the small intestine, we analyzed ileum tissue from pigs with extremely high and low feed efficiency. We identified numerous DELs, with 163 upregulated and 570 downregulated in the low RFI group. By combining these findings with differentially expressed genes, we identified 92 potential target genes. These genes are mainly involved in small molecule transport, metabolism, and lipid metabolism, indicating that lncRNAs primarily influence pig feed efficiency through metabolic pathways in the small intestine.

In our study, we identified 15 upregulated genes in the low RFI group that interact with 7 downregulated lncRNAs. These genes are primarily associated with functions related to fat digestion and absorption, the PPAR signaling pathway, and amino acid metabolism. Among them, fatty acid binding proteins (FABPs) play a crucial role in lipid transport, with intestinal FABP2 facilitating lipid transportation from the intestinal lumen into intestinal cells [41]. Apolipoprotein A1 contributes to the production of high-density lipoprotein particles essential for cholesterol reverse transport [42]. Sodium/glucose cotransporter 1 (SLC5A1) is responsible for glucose absorption and transport in the intestines [43], while StAR-related lipid transfer domain containing 4 (STARD4) is involved in maintaining cholesterol homeostasis [44].

The upregulation of these genes in the small intestine of pigs with low RFI suggests enhanced small molecule metabolism and transport capabilities, indicating a potential mechanism underlying improved feed efficiency in these animals. Furthermore, our findings align with previous studies by Wang et al [45] and Wu et al [46], which reported enrichment of genes and proteins related to membrane composition, transport processes, and intestinal motility pathways in pigs with divergent feed efficiency levels.

In the realm of lncRNA functionalities, two pertain to miRNA: lncRNA can act as a precursor for miRNA and can also function as a “sponge,” binding to miRNA to counteract their inhibitory effect on mRNA expression [23]. The ceRNA network offers insights into the regulatory crosstalk among lncRNAs, mRNAs, and miRNAs, elucidating potential regulatory mechanisms. Within this regulatory network, novel miRNAs have been unearthed, with LOC106504303 and LOC102160805 identified as potentially pivotal lncRNAs in the liver and ileum respectively. However, these potential interaction mechanisms warrant further exploration.

## CONCLUSION

In this study, we investigated the expression patterns of lncRNAs, miRNAs, and mRNAs in the liver and ileum of pigs with extreme feed efficiency. We identified DELs in the liver and ileum. These DELs are mainly involved in GTP metabolism and binding, nucleotide metabolism, catalytic enzyme activity, and transferase activity in liver, and small molecule transport, metabolism, and lipid metabolism in ileum. Meanwhile, LOC106504303 and LOC102160805 were identified as potentially key lncRNAs in the liver and ileum, respectively. These lncRNAs interact with miRNAs to regulate genes involved in mitochondrial oxidative phosphorylation and various ATP-related enzyme activities in the liver, as well as genes related to lipid and cholesterol metabolism, small molecule metabolism, and amino acid metabolism in the ileum. Overall, the lncRNAs regulate energy metabolism and biosynthesis in the liver, and the digestive absorption capacity in the small intestine, affecting the feed efficiency of pigs. However, further exploration is needed to fully understand the underlying mechanisms.

## Figures and Tables

**Table 1:** Animal performance of Duroc sows with highest and lowest RFI

Items	Low RFI (n = 10)	High RFI (n = 10)	p-value
RFI (kg)	−0.270±0.020	0.353±0.017	***
FCR	1.971±0.104	2.422±0.191	***
ADFI (kg/day)	1.863±0.125	2.358±0.190	***
ADG (kg/day)	0.946±0.067	0.975±0.052	NS
BFT (mm)	8.365±1.559	9.857±1.225	*

RFI, residual feed intake; FCR, feed conversion ratio; ADFI, average daily feed intake; ADG, average daily gain; BFT, back fat thicknesses, NS, not significant.

A p-value was calculated by t-test.

* p<0.05,

*** p<0.001.

**Table 2:** Significantly different lncRNA information in liver tissue

lncRNA	Log2FC	p-value	In LR group
BGIG9823_28378	3.733	0.005	Up
LOC110259032	3.661	0.011	Up
BGIG9823_28072	3.632	0.015	Up
LOC110255532	3.494	0.014	Up
LOC110261344	3.380	0.013	Up
BGIG9823_29867	3.366	0.024	Up
BGIG9823_42766	3.111	0.040	Up
BGIG9823_41210	3.110	0.026	Up
LOC102159985	2.995	0.022	Up
BGIG9823_30253	2.984	0.050	Up
BGIG9823_28921	−3.169	0.004	Down
LOC110257371	−3.173	0.034	Down
BGIG9823_37537	−3.215	0.001	Down
BGIG9823_47471	−3.255	0.021	Down
LOC110255870	−3.978	0.003	Down
BGIG9823_38767	−4.021	0.003	Down
LOC106505353	−4.089	0.003	Down
BGIG9823_35218	−4.303	0.002	Down
LOC110260319	−4.422	<0.001	Down
BGIG9823_37538	−4.779	<0.001	Down

lncRNA, long non-coding RNA; LR, low residual feed intake.

**Table 3:** Significantly different lncRNA information in ileum tissue

lncRNA	Log2FC	p-value	In LR group
LOC106504562	6.015	2.750E-06	Up
LOC110260443	5.665	7.306E-04	Up
LOC110261770	5.510	3.140E-06	Up
LOC102163428	5.384	4.795E-04	Up
LOC102160805	5.284	1.225E-04	Up
LOC110256029	5.047	4.650E-05	Up
LOC110259473	4.884	7.160E-06	Up
BGIG9823_29333	4.823	4.692E-04	Up
BGIG9823_38959	4.677	5.504E-04	Up
LOC102165887	4.525	1.565E-04	Up
BGIG9823_28387	−4.803	8.899E-04	Down
BGIG9823_46847	−4.839	1.570E-05	Down
BGIG9823_35290	−4.895	2.660E-03	Down
BGIG9823_43371	−4.911	1.371E-03	Down
BGIG9823_38708	−5.019	2.823E-03	Down
BGIG9823_29240	−5.109	1.165E-04	Down
LOC110255528	−5.354	1.557E-03	Down
BGIG9823_29766	−5.442	2.700E-05	Down
BGIG9823_31645	−5.467	1.513E-04	Down
BGIG9823_46019	−5.518	6.800E-06	Down

lncRNA, long non-coding RNA; LR, low residual feed intake.
